# MultiCapsNet: A General Framework for Data Integration and Interpretable Classification

**DOI:** 10.3389/fgene.2021.767602

**Published:** 2022-01-18

**Authors:** Lifei Wang, Xuexia Miao, Rui Nie, Zhang Zhang, Jiang Zhang, Jun Cai

**Affiliations:** ^1^ Shulan (Hangzhou) Hospital Affiliated to Zhejiang Shuren University Shulan International Medical College, Hangzhou, China; ^2^ China National Center for Bioinformation, Beijing, China; ^3^ Key Laboratory of Genomic and Precision Medicine, Beijing Institute of Genomics, Chinese Academy of Sciences, Beijing, China; ^4^ University of Chinese Academy of Sciences, Beijing, China; ^5^ School of Systems Science, Beijing Normal University, Beijing, China

**Keywords:** capsule network, classification, data integration, interpretability, modular feature

## Abstract

The latest progresses of experimental biology have generated a large number of data with different formats and lengths. Deep learning is an ideal tool to deal with complex datasets, but its inherent “black box” nature needs more interpretability. At the same time, traditional interpretable machine learning methods, such as linear regression or random forest, could only deal with numerical features instead of modular features often encountered in the biological field. Here, we present MultiCapsNet (https://github.com/wanglf19/MultiCapsNet), a new deep learning model built on CapsNet and scCapsNet, which possesses the merits such as easy data integration and high model interpretability. To demonstrate the ability of this model as an interpretable classifier to deal with modular inputs, we test MultiCapsNet on three datasets with different data type and application scenarios. Firstly, on the labeled variant call dataset, MultiCapsNet shows a similar classification performance with neural network model, and provides importance scores for data sources directly without an extra importance determination step required by the neural network model. The importance scores generated by these two models are highly correlated. Secondly, on single cell RNA sequence (scRNA-seq) dataset, MultiCapsNet integrates information about protein-protein interaction (PPI), and protein-DNA interaction (PDI). The classification accuracy of MultiCapsNet is comparable to the neural network and random forest model. Meanwhile, MultiCapsNet reveals how each transcription factor (TF) or PPI cluster node contributes to classification of cell type. Thirdly, we made a comparison between MultiCapsNet and SCENIC. The results show several cell type relevant TFs identified by both methods, further proving the validity and interpretability of the MultiCapsNet.

## Introduction

Recent advances in experimental biology have generated huge amounts of data. More detectable biological targets and various new measuring methods produce data at an unprecedented speed. For example, Microwell-Seq, a single cell RNA sequencing technology, has been used to analyze the transcriptome of more than 4,00,000 mouse single cells, covering all major mouse organs (Han et al., 2018); Single cell bisulfite sequencing (scBS-seq) has been designed to measure genome-wide DNA methylation at the single-cell level (Smallwood et al., 2014); and mass-spectrometry based technologies could explore the composition, structure, function, and control of the proteome (Aebersold and Mann, 2016). In addition, large and complex data sets are produced by large-scale projects, such as “The Cancer Genome Atlas” (TCGA) (Tomczak et al., 2015), and “Encyclopedia of DNA Elements” (ENCODE) (Consortium, 2004), which were established through community cooperation. There is an urgent need for next generation methods to deal with large, heterogeneous and complex data sets (Camacho et al., 2018).

As a promising data processing method, deep learning methods have been employed in biological data processing (Alipanahi et al., 2015; Camacho et al., 2018; Zhou et al., 2018; Eraslan et al., 2019). Various deep learning models could deal with various input data with different types and formats. For example, RNA sequence data as real-value vectors could be processed by simple feed forward neural network, which is a component of more complex models, such as auto-encoder (AE) (Lin et al., 2017; Chen et al., 2018), variational auto-encoder (VAE) (Ding et al., 2018), and Generative adversarial network (GAN) (Lopez et al., 2018). Sequence information, which is coded by ATCG, could be converted into real valued vectors by deep learning model using convolution neural networks (CNN) after model training (Alipanahi et al., 2015). Furthermore, deep learning models could integrate data with different types and formats. For example, DeepCpG utilizes both DNA sequence patterns and neighboring methylation states for predicting single-cell methylation state and modeling the sources of DNA methylation variability (Angermueller et al., 2017). However, the deep learning methods usually run as a “black box”, which is hard to interpret (Almas Jabeen and Raza, 2017). Great efforts have been made to improve the interpretability of deep learning models. The prior biological information, such as regulation between transcription factors (TF) and target genes or priori defined gene sets that retain the crucial biological features, could specify connections between neurons in the neural networks in order to associate the internal node (neuron) in the neural networks with TFs and thereby ease the difficulty of interpreting models (Lin et al., 2017; Chen et al., 2018). New probabilistic generative models with more interpretability, such as variational inference neural networks, are applied to scRNA-seq data for dimension reduction (Ding et al., 2018).

**FIGURE 1:**
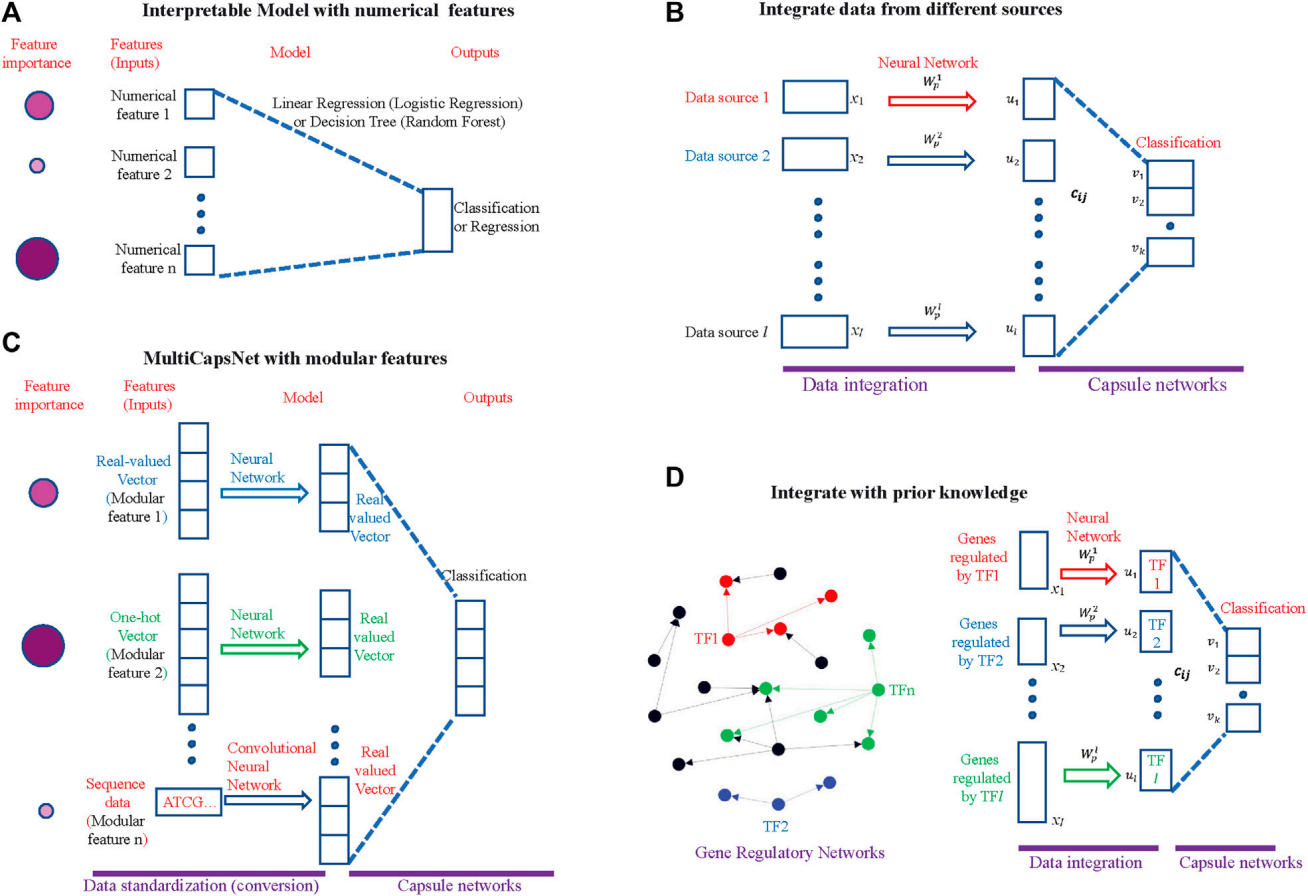
MultiCapsNet is an interpretable classifier and data integrator with modular inputs**. (A)** The traditional interpretable machine learning methods. The input of this model is numerical. After training, the model will reveal the inputs (features) importance for classification (or regression). The size and color depth of the circle indicate the importance of the features, while the larger and darker circle indicates that the feature is more important. **(B)** The MultiCapsNet is an interpretable classifier with modular input. The inputs (features) with different format (real-valued vector, one-hot encoding vector, or sequence data) and different lengths are first converted into real-valued vectors with equal length through trainable networks. Then, classification was based on those real-valued vectors of the same length. After training, the model will reveal the inputs (features) importance to classification. The size and color depth of the circle indicate the importance of the feature, while larger and darker circle indicates that the feature is more important. **(C)** The MultiCapsNet could integrate data from different sources. **(D)** The MultiCapsNet could integrate prior knowledge, such as gene regulatory information. Left: Gene regulatory networks, transcription factor and its targets are marked with same color. Right: expression of genes that are regulated by the same transcription factor could be regarded as a data source.

Traditional interpretable machine learning methods, such as linear regression (logistic regression) or decision tree (random forest), could only deal with numerical or categorical feature (Molnar, 2019) (Figure 1A). However, in the field of biology, especially in the field of network biology, the data is highly modular in nature. For example, in drug discovery, many independent features with multiple labels (e.g., response to drug, and disease state) across a multitude of data types (e.g., expression profiles, chemical structures) are needed; and in synthetic biology, the input may include sequence data, composition data and functional data (Camacho et al., 2018). An interpretable machine learning method adapted with modular input is demanded.

The capsule network (CapsNet) is a newly developed deep learning model for digital recognition tasks (Sabour et al., 2017). In the realm of biology, the CapsNet model has been directly applied for protein structure classification and prediction (Dan Rosa de Jesus et al., 2018; Fang et al., 2018) and is ripe for application in network biology and disease biology with data from multi-omics dataset (Camacho et al., 2018). In our previous work, we proposed a modified CapsNet model, called single cell capsule network (scCapsNet), which is suitable for single-cell RNA sequencing (scRNA-seq) data (Wang et al., 2020). The scCapsNet is a highly interpretable cell type classifier, with the capability of revealing cell type associated genes by model internal parameters.

Here, we introduce MultiCapsNet, a deep learning classifier and data integrator built on CapsNet and scCapsNet. As a general framework, the MultiCapsNet model should be able to deal with modular data from multiple sources with different formats and lengths, and give the importance scores of each data source for prediction after training (Figures 1B–D). In order to demonstrate its wide biological application, the MultiCapsNet model was tested on three data sets. In the first example, we applied the MultiCapsNet model to the labeled variant call data set, which was originally used to test the models for automating somatic variant refinement (Ainscough et al., 2018). According to data source and data attributes, the 71 features listed in the data set were divided into eight groups. Then the features in one group were viewed as a whole to train the MultiCapsNet model. After training, the performance of our MultiCapsNet matches well with the previous feed forward neural network model and random forest model. As an advantage our MultiCapsNet model directly provides the importance score for each data source, while the previous feed forward neutral network model needs an extra importance determination step through shuffling individual features to do so. Despite that our MultiCapsNet model is substantially different from the previous feed forward neural network model and the source importance measuring methods are also different, the correlation between the importance scores generated by those two models is highly correlated. In the second example, we demonstrate how to integrate prior knowledge and scRNA-seq data through MultiCapsNet model. The protein-protein interactions (PPI) information stored in BIOGRID (Stark et al., 2006) and HPRD (Keshava Prasad et al., 2009), and protein-DNA interactions (PDI) from DREM 2.0 (Schulz et al., 2012), are used as prior knowledge to specify network connections, as in previous work (Lin et al., 2017). In this example, the structures of the first part of the MultiCapsNet model, i.e., the connections between inputs and primary capsules, are determined by the PPI and PDI information. As a result of these specified structures, each primary capsule is labeled either as TF or PPI subnetwork (PPI), and inputs of each primary capsule could be regarded as a data source. We use data from mouse scRNA-seq dataset (Han et al., 2018) to train this MultiCapsNet model and the classification accuracy of MultiCapsNet is comparable to neural network and random forest model. After training, the MultiCapsNet model reveals how each primary capsule, which is labeled either as TF or PPI subnetwork (PPI), contributes to cell type classification. The top contributors of a particular cell type are usually related to that cell type. In the third example, we make a comparison between our MultiCapsNet and the established single-cell regulatory network inference method: SCENIC (Single-cell regulatory network inference and clustering) (Aibar et al., 2017). The results show that many cell types relevant TFs are identified by both methods, which further proves the validity and interpretability of MultiCapsNet.

## Methods

### Datasets and Data Preprocessing

Labeled variant call dataset from previous work was used to test the MultiCapsNet model (Ainscough et al., 2018). This dataset contains more than 41,000 samples, which are assembled to train models for automating somatic variant refinement. Each sample in the dataset is manually labeled as one of four tags by the reviewer: “somatic”, “ambiguous’, “germline”, and “fail”, which represent the confidence of a variant call by upstream somatic variant caller. As in previous work, we merged the variant calls labeled as “germline” and “fail” into a class named “fail”. The number of instances in each class are around 10,000, 13,000, 18,000 for “ambiguous”, “fail”, and “somatic”. There are 71 features that are associated with each sample, including cancer types, reviewers, tumor read depth, normal read depth, and so on. According to the data sources and data attributes, we divided these 71 features into eight groups (Supplementary Table S1). Group 1 contains nine cancer types, and is encoded as one-hot encoding vector. We call group 1 as “Disease” because it indicates the disease to which each variant call belongs. Group 2 contains four reviewers, and is encoded as one-hot encoding vector. We call group 2 as “Reviewer”. Group 3 contains information of “normal VAF”, “normal depth”, “normal other bases count”, and is called as “Normal_pro”, short for “Normal properties”. Group 4 contains 13 features that describe reference reads in normal, including base quality, mapping quality, numbers of mismatches, numbers of minus and plus strand, and so on. We call group 4 as “Normal_ref”. Group 5 contains 13 features extracted from variant reads in normal, also including base quality, mapping quality, numbers of mismatches, numbers of minus and plus strand, and so on. We call group 5 as “Normal_var”. The last three groups contain features drawn from tumor instead of normal in previous three groups. As same as Group 3, 4, and 5, we label group 6, 7, and 8 as “Tumor_pro”, “Tumor_ref”, and “Tumor_var” respectively.

The mouse scRNA-seq is measured by Microwell-Seq (Han et al., 2018). We downloaded scRNA-seq data and the annotation information through the link provided by the authors (https://figshare.com/s/865e694ad06d5857db4b). Then we use the annotation information to select parts of data from whole dataset. The cell types we chose include “Cartilage cell”, “Secretory alveoli cell”, “ Epithelial cell ”, “Kupffer cell”, “Muscle cell”, “Dendritic cell”, “ Spermatocyte”, and the number of instances in each cell type are 527, 1,195, 1,219, 356, 626, 717, 353. Moreover, we only use the genes contained in prior knowledge (Lin et al., 2017) to fit the model structure, and set the default value to zero when the downloaded scRNA-seq data does not contain this gene (Han et al., 2018).

A SCENIC example dataset was used to compare the performances of MultiCapsNet and SCENIC (https://scenic.aertslab.org/examples/). The dataset (sceMouseBrain.RData) contains seven cell types of mouse cortex and hippocampus (Zeisel et al., 2015) [“astrocytes_ependymal” (224), “endothelial_mural” (235), “interneurons” (290), “microglia” (98), “oligodendrocytes” (820), “pyramidal_CA1” (939), and “pyramidal_SS” (399)].

### The Architecture and Parameters of the MultiCapsNet Model

In the architecture of our multiCapsNet model, there are *l* neural networks corresponding to *l* input modular data.

$$u_{i}=\;\tanh\left(W_{p}^{i}x_{i}\right)\;\;\;\;\;\;i\in \left[1,2\ldots ,l\right]$$ (1)

*x*_*i*_ represents *i*’s input modular data. $W_{p}^{i}$ represents weight matrices of neural networks with dimension (*n, r*_*i*_), where the *r*_*i*_ is the length of the input modular data *x*_*i*_. The output $u_{i}$ of each neural network i (i∈[1,2…,l]) is a vector with length *n*, viewed as “primary capsule” in the model. The inputs standardization part converts the modular data with different type and length into real valued vectors with equal length n (*n* = 8 by default).

The standardized information is subsequently delivered through primary capsule to the capsule in the final layer by “dynamic routing” (Supplementary Figure S1). Each capsule in the final layer, named “type capsule”, corresponds to each cell type. They are denoted as vectors $v_{j}$ , where j∈[1,2…,k] , k is the number of cell types and *m* is the length of vectors. The capsule network module is implemented in Keras (https://github.com/bojone/Capsule).

Prior to the “dynamic routing” process, the primary capsules are multiplied by weight matrices $W_{ij}$ to produce “prediction vectors” ${\hat{u}}_{j|i}$ .

$${\hat{u}}_{j|i}\;=W_{ij}u_{i}$$ (2)

Then the iterative dynamic routing begins. Firstly, the “coupling coefficients” $c_{ij}$ is calculated by formula:

$$c_{ij\;}=\frac{\exp\left(b_{ij}\right)}{\sum_{k}\exp\left(b_{ik}\right)}$$ (3)

Where $b_{ij}$ is an intermediate parameter with initial value of zero, representing the inner product of the prediction vector and type capsule vector.

In order to compute the $b_{ij}$ for next round iteration, the weighted sum $s_{j}$ over all k prediction vectors ${\hat{u}}_{j|i}$ is calculated by formula:

$$s_{j}\;=\;normalize\left(\underset{i}{\sum }c_{ij}{\hat{u}}_{j|i}\right)$$ (4)

Secondly $b_{ij}$ is computed by the dot product of ${\hat{u}}_{j|i}$ and $s_{j}$ as the last step of one round dynamic routing process.

$$b_{ij}\;={\hat{u}}_{j|i}.s_{j}$$ (5)

After several rounds of dynamic routing, the type capsule $v_{j}$ is calculated by a non-linear “squashing” function:

$$v_{j}\;=\frac{{\left\|s_{j}\right\|}^{2}}{0.5+{\left\|s_{j}\right\|}^{2}}\frac{s_{j}}{\left\|s_{j}\right\|}$$ (6)

The following pseudocode illustrates the implementation of MultiCapsNet.

1) $for\;all\;primary\;capsule\;i:u_{i}=\;Activation\;Function\left(W_{p}^{i}x_{i}\right)$

2) for all primary capsule i  and type capsule *j*: ${\hat{u}}_{j|i}\;=W_{ij}u_{i}$

3) procedure ROUTING $\left({\hat{u}}_{j|i},r\right)$

4) for all primary capsule I and type capsule *j*: $b_{ij}\leftarrow 0$ .
5) For r iterations do
6) for all primary capsule *i*: $c_{i}$ ← softmax $\left(b_{i}\right)$

7) for all type capsule *j*: $s_{j}\;\leftarrow \;normalize\left(\underset{i}{\sum }c_{ij}{\hat{u}}_{j|i}\right)$

8) for all primary capsule *i* and type capsule *j*: $b_{ij}\;\leftarrow \;{\hat{u}}_{j|i}.s_{j}$

return $v_{j}\leftarrow$ squash ( $s_{j}$ )

The implementation of MultiCapsNet can be found in https://github.com/wanglf19/MultiCapsNet.

### MultiCapsNet Model in Somatic Variant Refinement Task

In the somatic variant refinement task, the eight groups mentioned above in the section of “Datasets and data preprocessing” correspond to eight input sources. Therefore, there are eight neural networks corresponding to eight groups of input modular data (*l = 8*).

$$u_{i}=\;\tanh\left(W_{p}^{i}x_{i}\right)\;\;\;\;\;i\in \left[1,2\ldots ,8\right]$$ (7)

**FIGURE 2:**
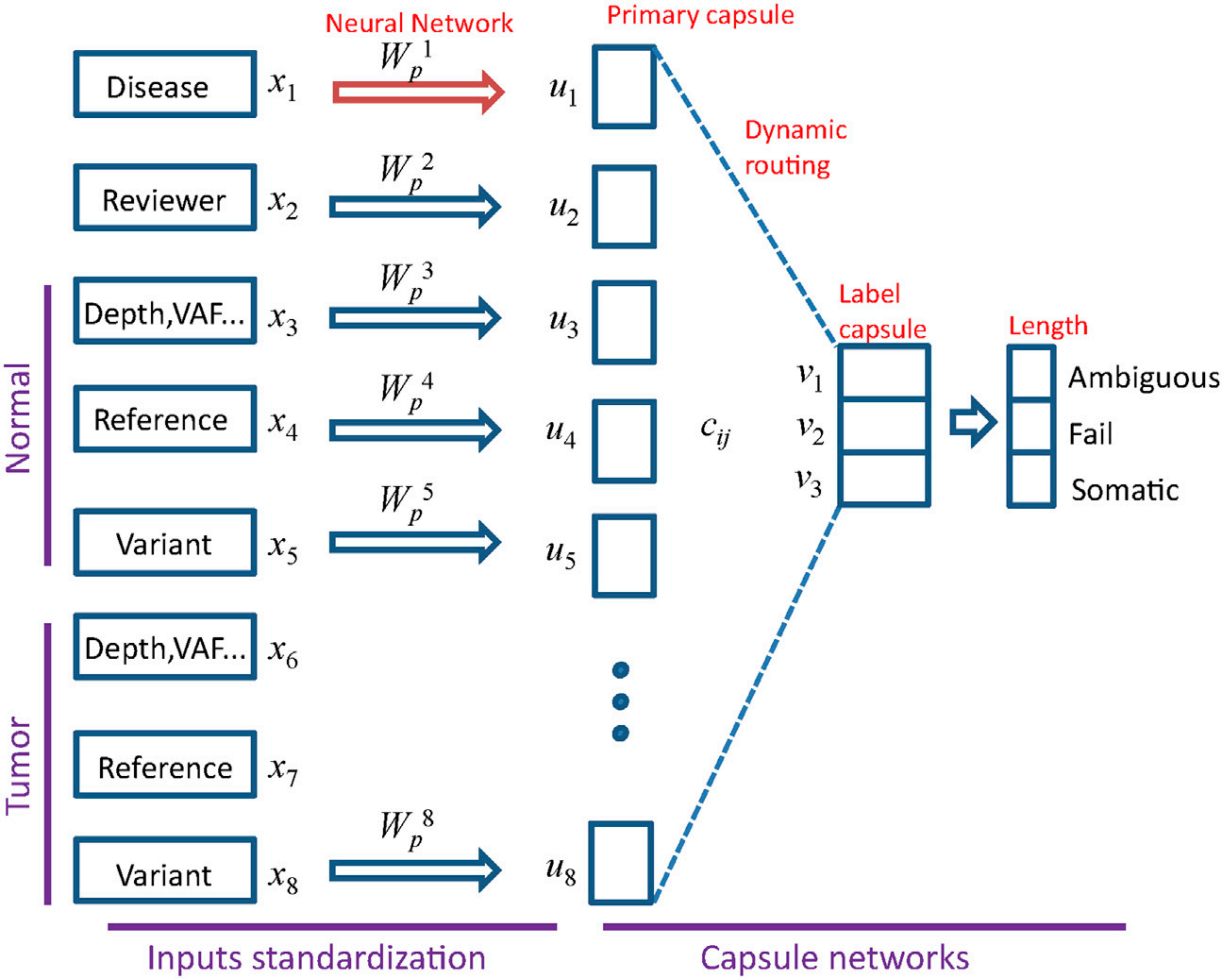
Architecture of MultiCapsNet with two layers. The first layer consists of eight parallel neural networks, corresponding to eight data sources (groups). The outputs of neural networks are the primary capsules (real valued vectors) with equal length. The second layer is the Keras implementation of CapsNet for classification. The length of each label capsule represents the probability that the input data belongs to the corresponding classification category.

After the input standardization part, the input data *x*_*i*_ is converted into a primary capsule *u*_*i*_ having the same length. Next, the standardized information stored in the primary capsules would be delivered to the final layer capsules by “dynamic routing”. The capsules in the final layer, which corresponds to labels of variant calls, is called “label capsule”. In capsnet, the non-linear “squashing” function ensure that short vectors get shrunk to almost zero length and long vectors get shrunk to a length slightly below 1 (Sabour et al., 2017). The length of the label capsule represents the probability that a variant call is either “ambiguous”,“fail”, or “somatic” (Figure 2). To evaluate the performance of the model, we use the “area under the curve” (AUC) score as previous (Ainscough et al., 2018) and prediction accuracy.

### MultiCapsNet Model That Integrates Prior Knowledge

The MultiCapsNet could integrate prior knowledge into its structure. In brief, PPI information store in BIOGRID (Stark et al., 2006) and HPRD (Keshava Prasad et al., 2009), and PDI coming from DREM 2.0 (Schulz et al., 2012), are used as prior knowledge for specifying network connections between the inputs and the primary capsules (Figure 4A), just as previous work used this prior knowledge to specify network connections between the inputs and neurons (Lin et al., 2017). For example, the prior knowledge indicates that Gene_1_,…, Gene_n_ are regulated by a TF (colored with green), so there are connections between Gene_1_,…, Gene_n_ and primary capsule representing corresponding TF (green connection); the prior knowledge indicate that Gene_2_,…, Gene_n_ are regulated by a TF (colored with blue), then there are connections between Gene_2_,…, Gene_n_ and primary capsule representing corresponding TF (blue connection); and the prior knowledge indicates that Gene_2_, Gene_3_,…, are in a subnetwork of PPI network (colored with red), then there are connections between Gene_2_, Gene_3_,…, and primary capsule representing corresponding PPI subnetwork (red connection). Although there is only one input source, namely scRNA-seq data, the input source can be decomposed into several parts by integrating prior knowledge, and each part is connected to a primary capsule. Therefore, we also took a single input source integrated with prior knowledge as an input from multiple sources, each of which is associated with a TF or a PPI subnetwork (Figure 4B).

In total there are 696 input modular data, with 348 TF-targets relationships extracted from PDI information and 348 PPI subnetworks. Therefore, there are 696 neural networks corresponding to 696 modular data (*l = 696*).

$$u_{i}=\tanh\left(W_{p}^{i}x_{i}\right)\;\;\;\;\;i\in \left[1,2\ldots ,696\right]$$ (8)

After the input standardization part, the input data *x*_*i*_ is converted into primary capsule *u*_*i*_ with the same length. Next, the standardized information stored in the primary capsules would be delivered to the final layer capsules by “dynamic routing”. The capsules in the final layer, which correspond to cell types, is called “type capsule”.

### MultiCapsNet Model Compared with SCENIC

The SCENIC is a workflow for simultaneous reconstruction of gene regulatory networks and identification of cell states using scRNA-seq data (Aibar et al., 2017). The workflow consists of three modules (R/bioconductor packages): GENIE3 (GRNboost), RcisTarget, AUCell. The first two modules were responsible to find potential TF-targets relationships based on co-expression and subsequently select the highly confident TF-target regulation according to TF-motif enrichment analysis. After that, several potential TF-target relationships across all cell types, called regulons, were identified in the dataset. The AUCell would score the activity of these regulons in each single cell. Finally, the unsupervised method is used to cluster cell, identify cell types and states based on the scores of the regulongs, which are used as features for each cell. In our model, we utilized the regulon information identified by the first two modules of SCENIC as the prior knowledge to specify the connections between input and primary capsules (Supplementary Figure S2A). The dataset, intermediate results and the output of SCENIC for a mouse brain example were downloaded from the website (https://scenic.aertslab.org/examples/). The regulon information was extracted from the intermediate result file (regulons_asGeneSet.Rds).

In total there are 253 regulons, which specify TFs and their target genes. Therefore, there are 253 neural networks corresponding to 253 modular data (*l =* 253).

$$u_{i}=\tanh\left(W_{p}^{i}x_{i}\right)\;\;\;\;\;i\in \left[1,2\ldots ,253\right]$$ (9)

After the input standardization part, the input data *x*_*i*_ is converted into primary capsule *u*_*i*_ with same length. Next, the standardized information stored in the primary capsules would be delivered to the final layer capsules by “dynamic routing”. The capsules in the final layer, which correspond to cell types, is called “type capsule”.

### Average Coupling Coefficients and Data Source Importance

In scCapsNet, we showed that the average coupling coefficients represent the contribution of the primary capsules to the final layer type capsules for each cell type (Wang et al., 2020). Similarly, in the multiCapsNet model, the type (label) capsule $v_{j}$ derives from a weighted sum of prediction vectors ${\hat{u}}_{j|i}$ . The weights are the coupling coefficients $c_{ij}$ and the magnitude of these coefficients could roughly be regarded as the contribution of the primary capsules $u_{i}$ to the type capsules $v_{j}$ . Each sample (single cell, somatic variant) generates its own coupling coefficients. The average coupling coefficients for samples with same type (label) are calculated by the formular:

$$c_{ij}^{type\;average}\;=\;\frac{\sum_{type}c_{ij}^{type}}{\sum_{type}1\;}$$ (10)

Therefore, each classification category (cell type/variant call label) corresponds to an average coupling coefficients matrix ( $c_{ij}^{type\;average}$ ), called type average coupling coefficients, with rows representing type capsules and columns representing primary capsules. The type average coupling coefficients matrix could be plotted as heatmap for visualization of data. For each classification category (cell type/variant call label), the corresponding type average coupling coefficients matrix contain an effective type capsule row, which is the row whose type is consistent with this classification category. For example, the effective type capsule row in the type average coupling coefficients matrix ( $c_{ij}^{type\;2\;\;average}$ ) is the row $c_{i2}^{type\;2\;\;average}$ . In this row, the magnitude of each element could be regarded as the importance score of the corresponding primary capsule to this classification category. The effective type capsule rows of all classification categories ( $c_{i1}^{type\;1\;\;average},\;\;c_{i2}^{type\;2\;\;average},\;\;c_{i3}^{type\;3\;\;average}$ …) could be organized into a new matrix, visually represented as an overall heatmap.

### Algorithm Implementation for Comparisons

A neural network with sigmoid activation function was implemented in Keras. The random forest and nearest-neighbour are implemented with the Python package “scikit-learn”. The comparison transformers model was originally used for IMDB movie review sentiment classification dataset. This transformer model contains the embedding layer for embedding the words into vectors and the Multi-head attention layer (https://github.com/bojone/attention/). We replace the embedding layer with our data standardization layer, and retain the Multi-head attention layer for classification.

## Results

### MultiCapsNet Achieves High Classification Accuracy and High Interpretability for Modular Data From Variant Call Dataset

The variant call dataset (Please refer to Datasets and data preprocessing in METHODS section for the details) was randomly divided into training set and validation set with a ratio of 9:1. Our MultiCapsNet model performs well in the classification of variant call (Figure 4). The results show that the AUC of the MultiCapsNet model is 0.94, 0.99, and 0.97, respectively, in the classification categories of “ambiguous”, “fail”, and “somatic” (Figure 4A). These AUC scores are similar with those obtained by the Multi-head Attention model (0.93, 0.98, 0.96), feed forward neural network (0.93, 0.99, 0.96), and random forest (0.96, 0.99, 0.98) (Ainscough et al., 2018). Meanwhile, the average prediction accuracy of the MultiCapsNet model is around 0.873, similar to those obtained by the Multi-head Attention model (0.866), and slightly lower than that of feed forward neural network (0.887), and random forest (0.895).

**FIGURE 3:**
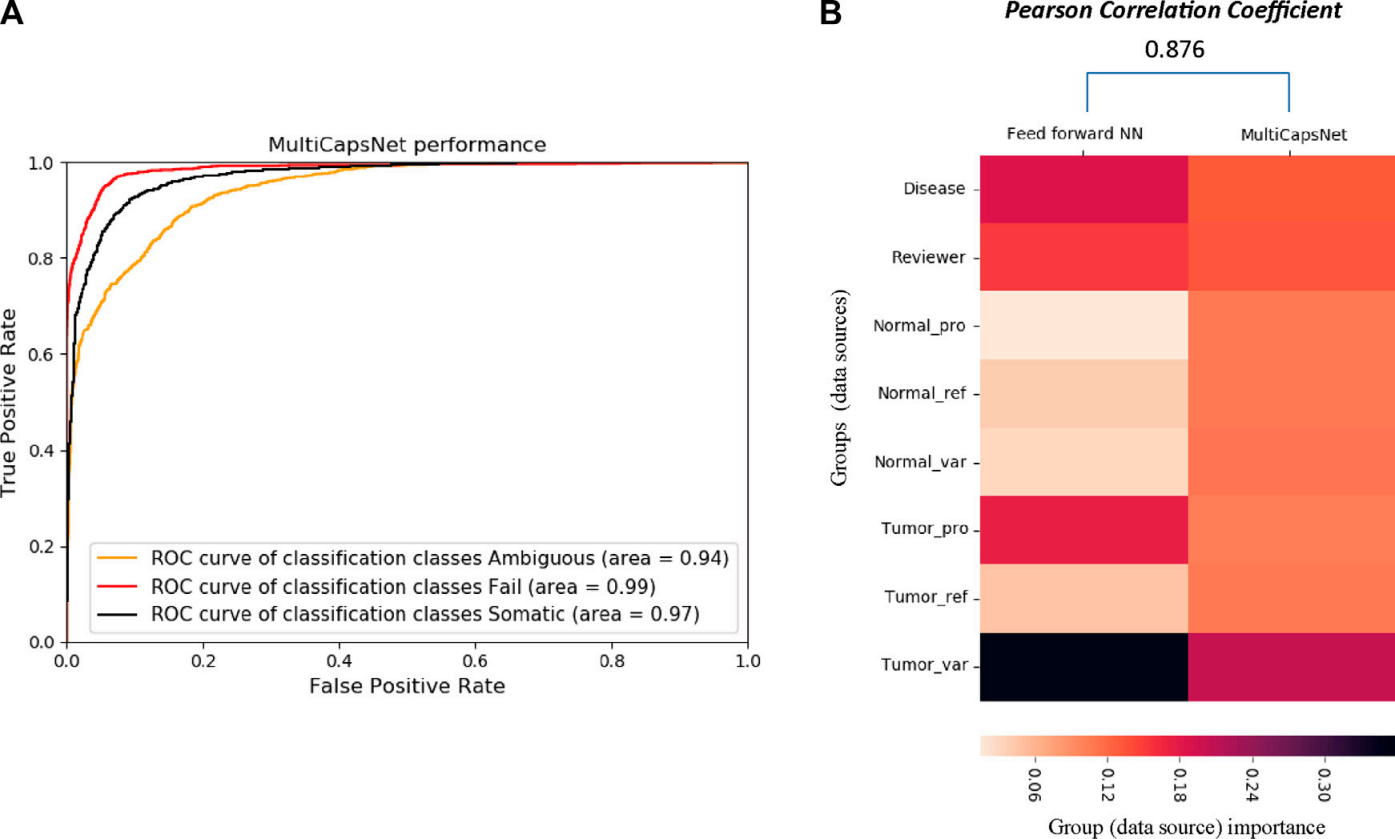
Architecture of MultiCapsNet integrated with prior knowledge. **(A)** The model has two layers. The first layer consists of 696 parallel neural networks corresponding to 696 primary capsules labeled with either transcription factor (348) or protein-protein interaction cluster node (348). The inputs of each primary capsule include genes regulated by a transcription factor or in a protein-protein interactions sub-network. The second layer is the Keras implementation of CapsNet for classification. The length of each final layer type capsule represents the probability of input data belonging to the corresponding classification category. **(B)** Alternative representation of MultiCapsNet integrated with prior knowledge. Genes that are regulated by a transcription factor or in a protein-protein interactions sub-network, are groups together as a data source for MultiCapsNet. Figures 3A,B are equivalent with different representation.

**FIGURE 4:**
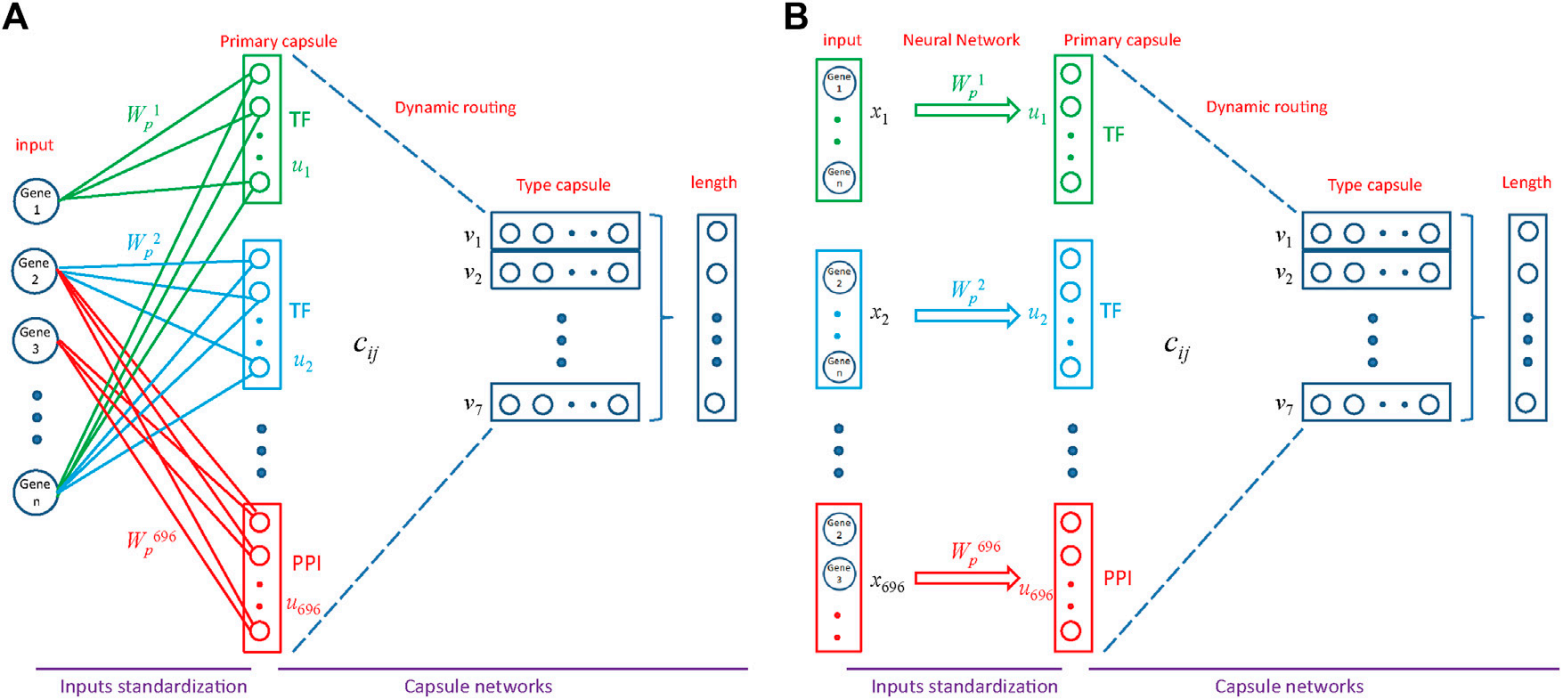
The comparison between MultiCapsNet and feed forward neural network shows the high performance and interpretability of MultiCapsNet. **(A)** The AUC scores demonstrate that the MultiCapsNet model achieves very high classification performances in all three classification categories. **(B)** The normalized group (data source) importance scores generated by MultiCapsNet and feed forward neural network are highly correlated.

In MultiCapsNet, the coupling coefficient *c*_*ij*_ is viewed as important scores, which is the weight that measure the contribution of each primary capsule to the final layer type capsule. Each input would generate its own coupling coefficient, and the type average coupling coefficient is the average over all the inputs with same classification category. After MultiCapsNet model training, the type average coupling coefficients for each variant label (“ambiguous”, “fail”, and “somatic”) were calculated and visualized as heatmaps (Supplementary Figure S3A) (Please refer to “METHODS” section for the detailed calculation formula of type average coupling coefficients). In each type average coupling coefficient, the most important row, named as “effective type capsule row”, is the row whose type is consistent with this classification category. The overall heatmap is assembled with the “effective type capsule row” which describes the importance scores of all the data sources for distinct category classification (Supplementary Figure S3B). Therefore, the overall heatmap also shows the contribution of each data source to the recognition of each variant labels (“ambiguous”, “fail”, and “somatic”). For example, the data source of “Disease” has the contribution to the classification of “somatic” category and the “Reviewer” source contributes to the classification of “ambiguous” category. The “Tumor_var” source is the most important one for the classification of all the three categories (Supplementary Figure S3B). Over 9 repetitions, the values of each row in 9 overall heatmap are averaged to determine the importance scores of each data sources for the classification of all the categories in MultiCapsNet model (Figure 3B). In feed forward neural network model, the feature importance is measured by average change of AUC after randomly shuffling individual features. Based on the step of features grouping, we added the feature importance scores belonging to the same group together, and take these values as importance of data sources (each group) in feed forward neural networks model (Figure 3B). Then, we calculated the correlation between the data source importance scores obtained by our MultiCapsNet model and those provided by feed forward neural network model. Although our MultiCapsNet model is substantially different from the previous feed forward neural network, and the source importance measuring methods are also different, there is very high correlation between them (Pearson Correlation Coefficient = 0.876) (Figure 4B). Both models indicate that tumor variant group is very important for variant call classification.

### MultiCapsNet Integrated with Prior Knowledge Could Function as Classifier and Identify Cell Type Relevant TF

**FIGURE 5:**
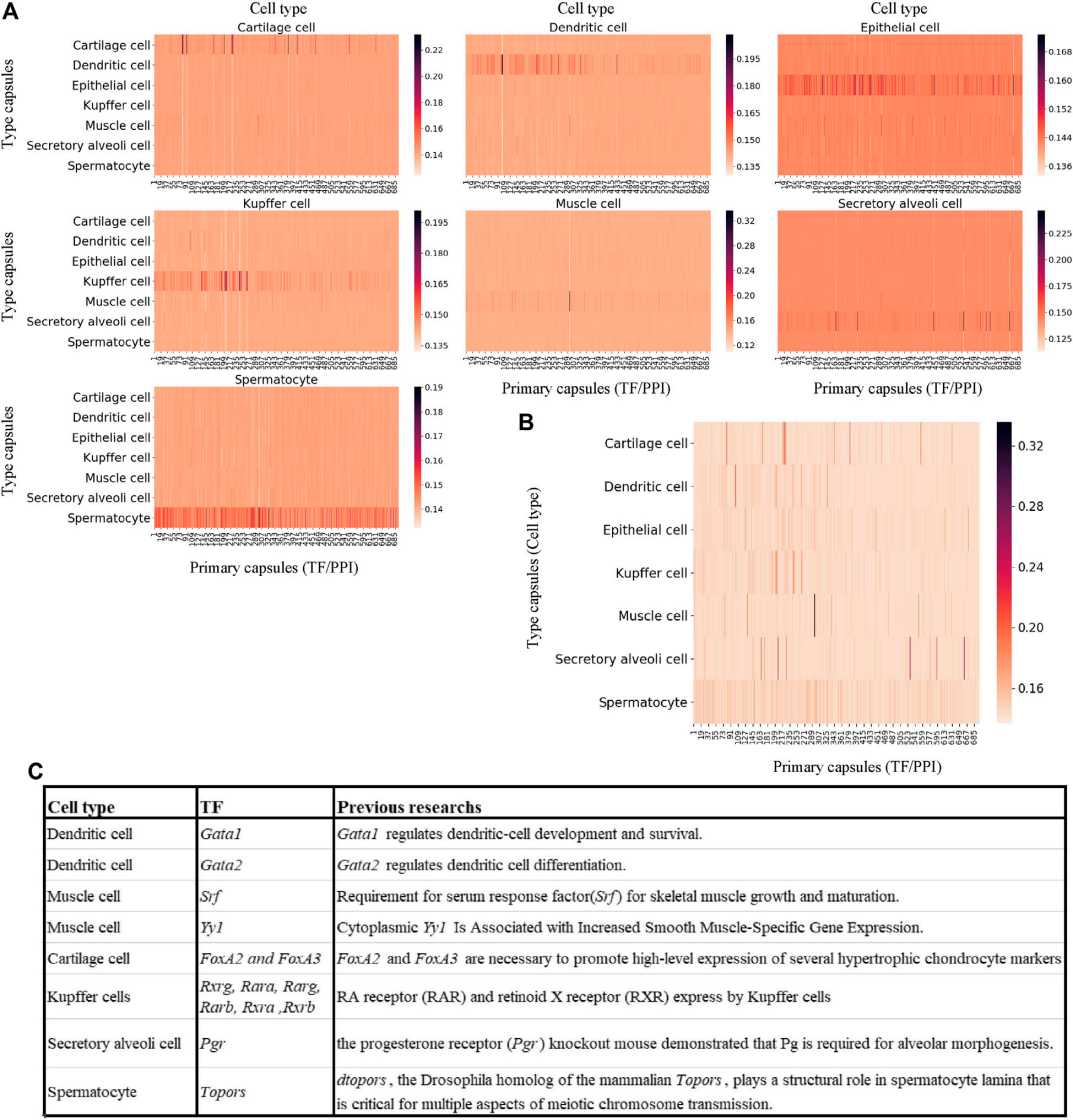
MultiCapsNet integrated with prior knowledge could identify cell type associated transcription factor. **(A)** heatmaps of the matrices of average coupling coefficients for each cell type. In each heatmap, there are 696 columns for 696 primary capsules (TF/PPI) and seven row for seven type capsules (cell types), and each element in the average coupling coefficients is represented by a thin line. The brightness of these thin lines (elements in the average coupling coefficients) indicate the contribution of the primary capsules (TF/PPI) to the specific cell type recognition. The dark lines (high score elements in average coupling coefficients) exclusively reside in the corresponding effective type capsule row in each heatmap. **(B)** Overall heatmap of the combined matrix of average coupling coefficients. The combined matrix contains the effective type capsule rows in Figure 5A where its recognition type is in accordance with the type of single cells input. **(C)** The table list several top ranked contributors for specific cell type recognition, given by the MuiltCapsNet model, are associated with corresponding cell types which have been reported before.

The dataset is a portion of mouse scRNA-seq data measured by Microwell-Seq, which consists of nearly 5,000 cells of seven types and 9,437 genes (Please refer to METHODS section for the details). The MultiCapsNet model that integrates prior knowledge (Figure 4) was trained and tested by using this dataset. The average validation accuracy and F1 score are around 97%, comparable with those generated by the feed forward neural network, Multi-head Attention model and random forest (Supplementary Figures S4A, B). After training, the average coupling coefficients, which represent the contribution of the primary capsules (TF/PPI) to the type capsules (Cell type), were calculated and visualized as heatmaps for each cell type (Figure 5A). In each heatmaps, we should clearly observe that the high value elements in the average coupling coefficients (dark line in the plot) are exclusively located in the effective type capsule row. Then, the corresponding type capsule row was selected from each heat map in Figure 5A, and organized into an overall heatmap (Figure 5B).

We repeat the training process 9 times and generate nine overall heatmaps accordingly. Based on the average value of the nine overall heatmaps, the top 10 relevant TFs/PPI subnetwork was generated (Supplementary Table S2). Most of the top 10 relevant TFs/PPI subnetwork were specific to one cell type, and many of them have been reported to be associated with corresponding cell types previously (Figure 5C). For example, *Gata1* and *Gata2* are top contributors for dendritic cell recognition. Previous work indicated that *Gata1* regulates dendritic cell development and survival (Gutiérrez et al., 2007), *Gata2* regulates dendritic cell differentiation (Onodera et al., 2016). *Srf* and *Yy1* are ranked as the top contributors for muscle cell recognition by the model. However, *Srf* is required for skeletal muscle growth and maturation (Li et al., 2005), *Yy1* is associated with increased smooth muscle specific gene expression (Favot et al., 2005). *FoxA2* and *FoxA3* are ranked as top contributors for Cartilage cell recognition, and *FoxA2* and *FoxA3* are necessary to promote high-level expression of several hypertrophic chondrocyte markers (Ionescu et al., 2012). The model reports *Rxrg*, *Rara*, *Rarg*, *Rarb*, *Rxra*, and *Rxrb* as top contributors for Kupffer cell recognition. Previous research report RA receptor (RAR) and retinoid X receptor (RXR) were expressed by Kupffer cells (Ulven et al., 1998; Ohata et al., 2000). *Pgr* is ranked as a top contributor for secretory alveoli cell recognition, and the progesterone receptor (*Pgr*) knockout mouse demonstrated that Pg is required for alveolar morphogenesis (Oakes et al., 2006). *Topors* is ranked as a top contributor for spermatocyte recognition. Previous work indicates *dtopors*, the *Drosophila* homolog of the mammalian *Topors*, plays a structural role in spermatocyte lamina that is critical for multiple aspects of meiotic chromosome transmission (Matsui et al., 2011).

### The Comparison of MultiCapsNet Model with SCENIC Shows That Several Cell Type Relevant TFs Are Identified by Both Methods

To further demonstrate the effectiveness of our MultiCapsNet model to reveal cell type related TFs from scRNA-seq data, we compare it with established single-cell regulatory network inference methods: SCENIC (Single-cell regulatory network inference and clustering) (Supplementary Figure S2A). The scRNA-seq data from mouse cortex and hippocampus were used to evaluate these two methods (Please refer to METHODS section for the details).

**FIGURE 6:**
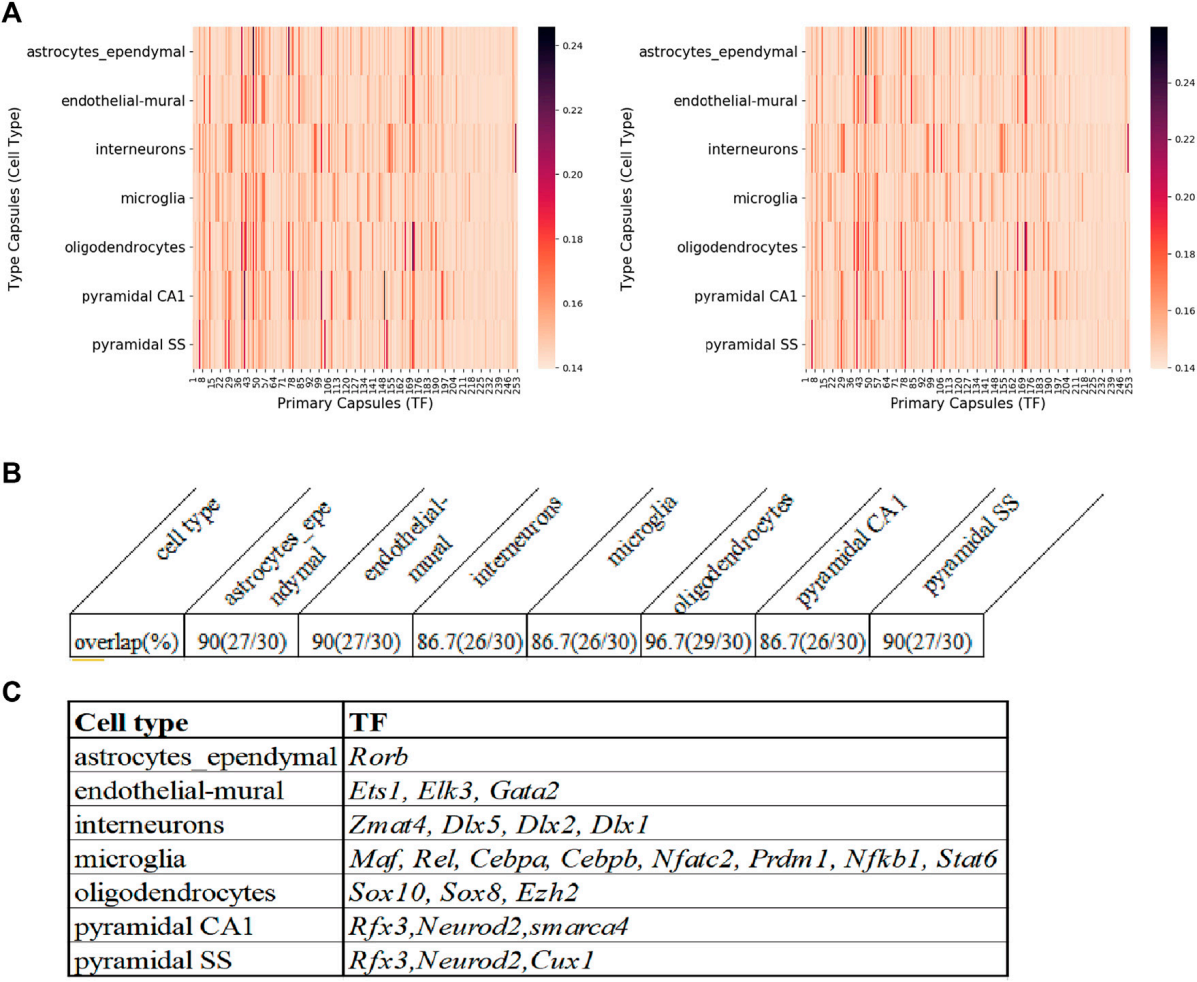
The comparison of MultiCapsNet and SCENIC shows the robustness and interpretability of MultiCapsNet. **(A)** Averaged overall heatmaps for mouse cortex and hippocampus dataset show that MultiCapsNet perform consistently whether including**(left)** or excluding **(right)** the labelling TF from regulon. **(B)** The top ranked contributors for specific cell type classification identified from dataset either including **(left)** or excluding **(right)** the labelling TF are highly overlapped. **(C)** The table list several top ranked contributors for specific cell type recognition, given by both the MuiltCapsNet model and SCENIC.

After MultiCapsNet training, the average coupling coefficients in the overall heatmap would indicate the most relevant TFs associated with each cell type (Supplementary Figure S5). We repeated the experiment 9 times, the average validation accuracy was 97%, and the average F1 score was around 95%, which were comparable to the results generated by feed forward neural network, Multi-head Attention model and random forest (Supplementary Figures S4C, D). According to the average value of nine overall heatmaps, the top 30 relevant TFs could be generated (Figure 6A left; Supplementary Table S3 top). The original regulon may contain TFs that label the 253 regulons. In order to eliminate the influence caused by the expression of those labeling TF, the potential TF-target relationships that exclude the labeling TF in the set of target genes are also made (Supplementary Figure S2B). We also repeated the training process of MultiCapsNet that integrated with those new potential TF-target relationships. After training, the top 30 relevant TFs could also be generated according to the average value of the nine overall heatmaps (Figure 6A right; Supplementary Table S3 bottom). The results show that the inclusion or exclusion of labeling TF has little influence on prediction accuracy and interpretability of the model. The overlap rates of top 30 most relevant TF of each cell type (around top 10% of total TFs) between model including labeling TF and that excluding labeling TF are very high, around 90% for every cell type (Figure 6B).

Many high score TFs predicted by MultiCapsNet are consistent with that reported by SCENIC (Aibar et al., 2017). For example, in both methods, *Rorb* is identified as a relevant TF for astrocytes; *Ets1*, *Elk3*, and *Gata2* are identified as relevant TFs for endothelial-mural cells; *Zmat4*, *Dlx5*, *Dlx2*, and *Dlx1* are identified as relevant TFs for interneurons; *Maf*, *Rel*, *Cebpa*, *Cebpb*, *Nfatc2*, *Prdm1*, *Nfkb1*, and *Stat6* are identified as relevant TFs for microglia; *Sox10* and *Sox8* are identified as relevant TFs for oligodendrocytes. Besides the TFs listed above, MultiCapsNet also detected several high confidence cell type relevant TFs that are also found by SCENIC. For example, *Rfx3* shows a high association with both pyramidal SS and CA1 cells. Previous studies reported that downstream target of *Rfx3* displayed cytosolic expression in pyramidal neurons (Remnestål, 2015) and *Rfx3* expresses in cortical pyramidal neurons (Benadiba et al., 2012). *Neurod2* is also identified as a relevant TF for both pyramidal SS and CA1 cells. Previous studies reported that *Neurod2* coordinates synaptic innervation and cell intrinsic properties to control excitability of cortical pyramidal neurons (Chen et al., 2016). *Cux1* has been identified as a relevant TF for pyramidal SS cells, and *Cux1* has been reported as a restricted molecular marker for the upper layer (II-IV) pyramidal neurons in murine cerebral cortex (Li et al., 2010). *smarca4* has been identified as relevant TF for pyramidal CA1 cells, and *Brg1/smarca4* deficiency leads to mouse pyramidal neuron degeneration (Deng et al., 2015). *Ezh2* has been suggested as a relevant TF for oligodendrocytes, and the expression of *Ezh2* in OPCs (oligodendrocytes precursor cells), even up to the stage of pre-myelinating immature oligodendrocytes, remains high (Copray et al., 2009) (Figure 6C). Furthermore, the MultiCapsNet found that *Rpp25* is strongly associated with interneurons which SCENIC did not, and *Rpp25* has been reported up-regulated in GABAergic interneuron (Fukumoto et al., 2018).

## Discussion

In the first example, we demonstrated that the proposed MultiCapsNet model performed well in the variant call classification. Data sources with different data types, such as one-hot encoding vector and real valued vectors, could be standardized into equal length vectors as primary capsules, and then pass the information into final layer capsules by dynamic routing. The importance of the data sources was measured by the sum of the overall average coupling coefficients as the co-product of the model training. These importance scores are highly correlated with the importance scores calculated by feed forward neural network, which are measured by average change in the AUC after randomly shuffling individual features.

In the second example, we incorporated PPI and PDI information into the structure of the MultiCapsNet model. This specified structure decomposed the input scRNA-seq data into several parts, each part corresponding to a group of genes regulated by a TF or from a protein interaction sub-network. Therefore, each part of the decomposition input was regarded as a data source, and the associated primary capsule could be marked as corresponding TF or PPI subnetwork. Although the number of the primary capsules was one order of magnitude more than that of previous CapsNet model, the model performed well, and its classification accuracy was comparable with those generated by feed forward neural network and random forest. After training, the contributions of each primary capsule and its corresponding data source to the cell type recognition were revealed by the MultiCapsNet model as co-product of classification. The TF or the PPI subnetwork that labeled the top ranked contributors were often relevant to the cell type they contributed. The comparison of our MultiCapsNet model with SCENIC showed several cell type relevant TFs identified by both methods, which further proves the validity and interpretability of the MultiCapsNet model.

To sum up, our MultiCapsNet model could integrate multiple input sources and standardize the inputs, then use the standardized information for classification through capsule network. In the variant call classification example, the data types are limited to one-hot encoding vectors or real valued vectors. With appropriate dataset, the MultiCapsNet could integrate and standardize more data types, such as sequence data, which can be integrated through convolutional neural network. In addition, our MultiCapsNet model could also incorporate the prior knowledge through adjusting the connection between layers according to the specification of the prior knowledge. In the example of scRNA-seq, we include only PPI and PDI information. In the future, the complex and hierarchical information of biological network will be introduced into the MultiCapsNet model to better understand the intricacies of disease biology (Camacho et al., 2018). Compared with other interpretable machine learning methods, MultiCapsNet could obtain similar classification accuracy under the condition of modular inputs, making it more suitable for the modular biological data.

MultiCapsNet model provides a framework for data integration, especially for multi-omics datasets, which have data from different sources and with different types and formats, or require prior knowledge. Once the data could be transformed into real valued vectors through trainable parameters, the data and transformation process could be integrated into the MultiCapsNet model as a building block. In this sense, the MultiCapsNet model possesses enormous flexibility, and is applicable in many scenes, let alone that it can measure the importance of data sources accompanying the training step without any extra calculation step.

## Data Availability

Publicly available datasets were analyzed in this study. This data can be found here: https://github.com/wanglf19/MultiCapsNet.
