# Corrigendum: MultiCapsNet: A General Framework for Data Integration and Interpretable Classification

**DOI:** 10.3389/fgene.2021.822045

**Published:** 2022-01-27

**Authors:** Lifei Wang, Xuexia Miao, Rui Nie, Zhang Zhang, Jiang Zhang, Jun Cai

**Affiliations:** ^1^ Shulan (Hangzhou) Hospital Affiliated to Zhejiang Shuren University Shulan International Medical College, Hangzhou, China; ^2^ China National Center for Bioinformation, Beijing, China; ^3^ Key Laboratory of Genomic and Precision Medicine, Beijing Institute of Genomics, Chinese Academy of Sciences, Beijing, China; ^4^ University of Chinese Academy of Sciences, Beijing, China; ^5^ School of Systems Science, Beijing Normal University, Beijing, China

**Keywords:** capsule network, classification, data integration, interpretability, modular feature

In the original article, there was a mistake in the number labeling for Figure 3 and Figure 4 as published. Figure 3 should be labeled as Figure 4, and vice versa. The correct legend appears below.

**FIGURE 3:**
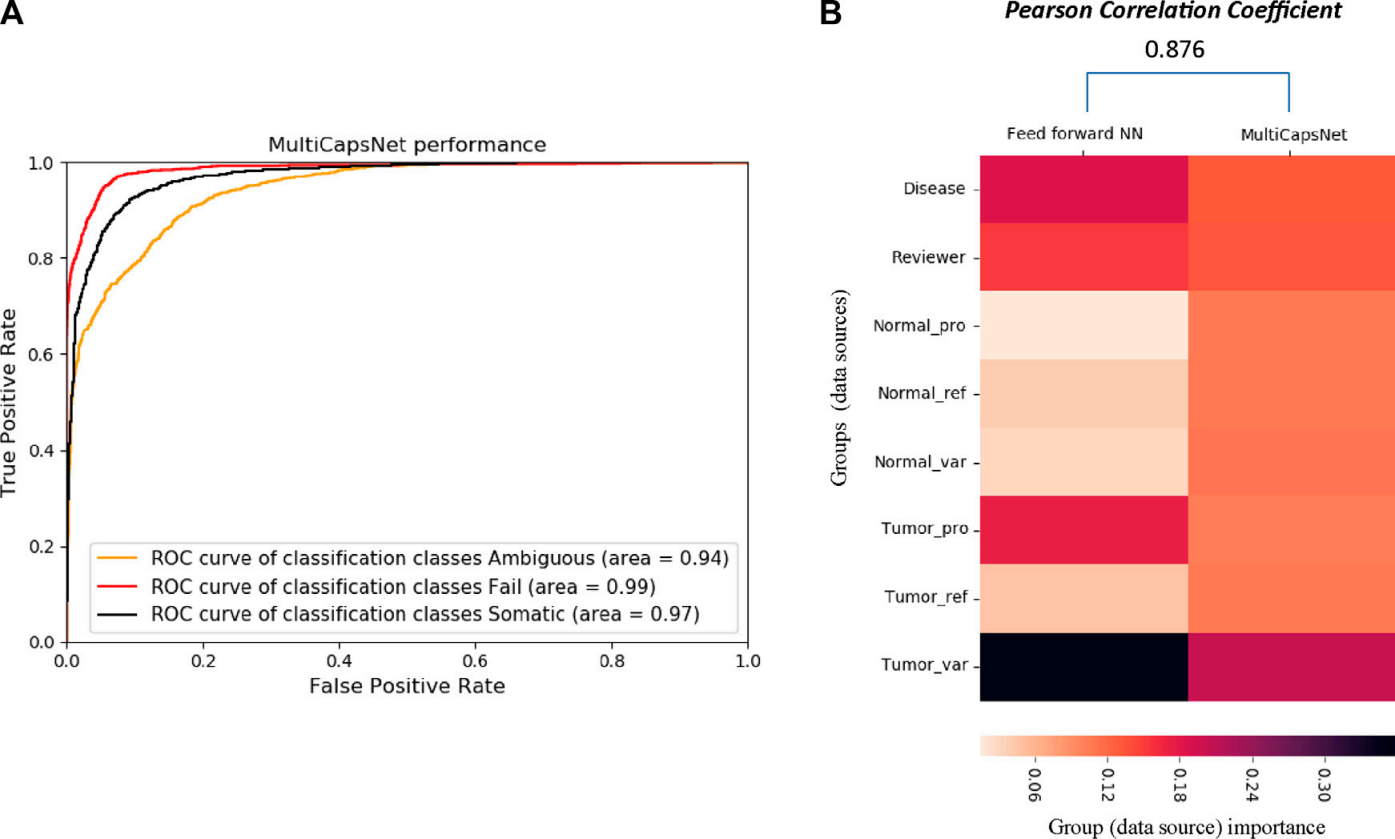
Architecture of MultiCapsNet integrated with prior knowledge. **(A)** The model has two layers. The first layer consists of 696 parallel neural networks corresponding to 696 primary capsules labeled with either transcription factor (348) or protein-protein interaction cluster node (348). The inputs of each primary capsule include genes regulated by a transcription factor or in a protein-protein interactions sub-network. The second layer is the Keras implementation of CapsNet for classification. The length of each final layer type capsule represents the probability of input data belonging to the corresponding classification category. **(B)** Alternative representation of MultiCapsNet integrated with prior knowledge. Genes that are regulated by a transcription factor or in a protein-protein interactions sub-network, are groups together as a data source for MultiCapsNet. Figures 3A,B are equivalent with different representation.

**FIGURE 4:**
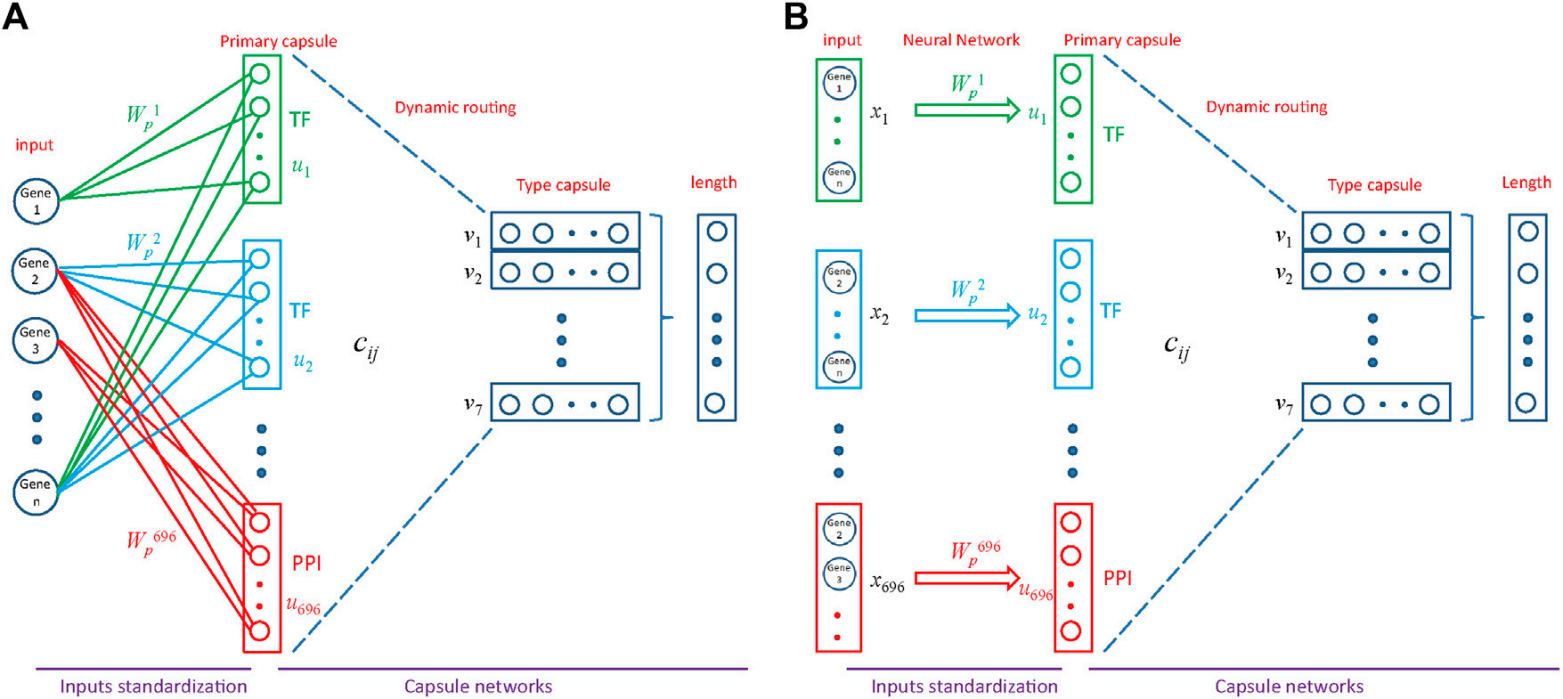
The comparison between MultiCapsNet and feed forward neural network shows the high performance and interpretability of MultiCapsNet. **(A)** The AUC scores demonstrate that the MultiCapsNet model achieves very high classification performances in all three classification categories. **(B)** The normalized group (data source) importance scores generated by MultiCapsNet and feed forward neural network are highly correlated.

The authors apologize for this error and state that this does not change the scientific conclusions of the article in any way. The original article has been updated.

