# Supplementary figures and images for: MultiCapsNet: A General Framework for Data Integration and Interpretable Classification
Source: Front Genet. 2022 Jan 18;12:767602. doi: 10.3389/fgene.2021.767602 (PMC8652257; doi:10.3389/fgene.2021.767602)

Figure S1

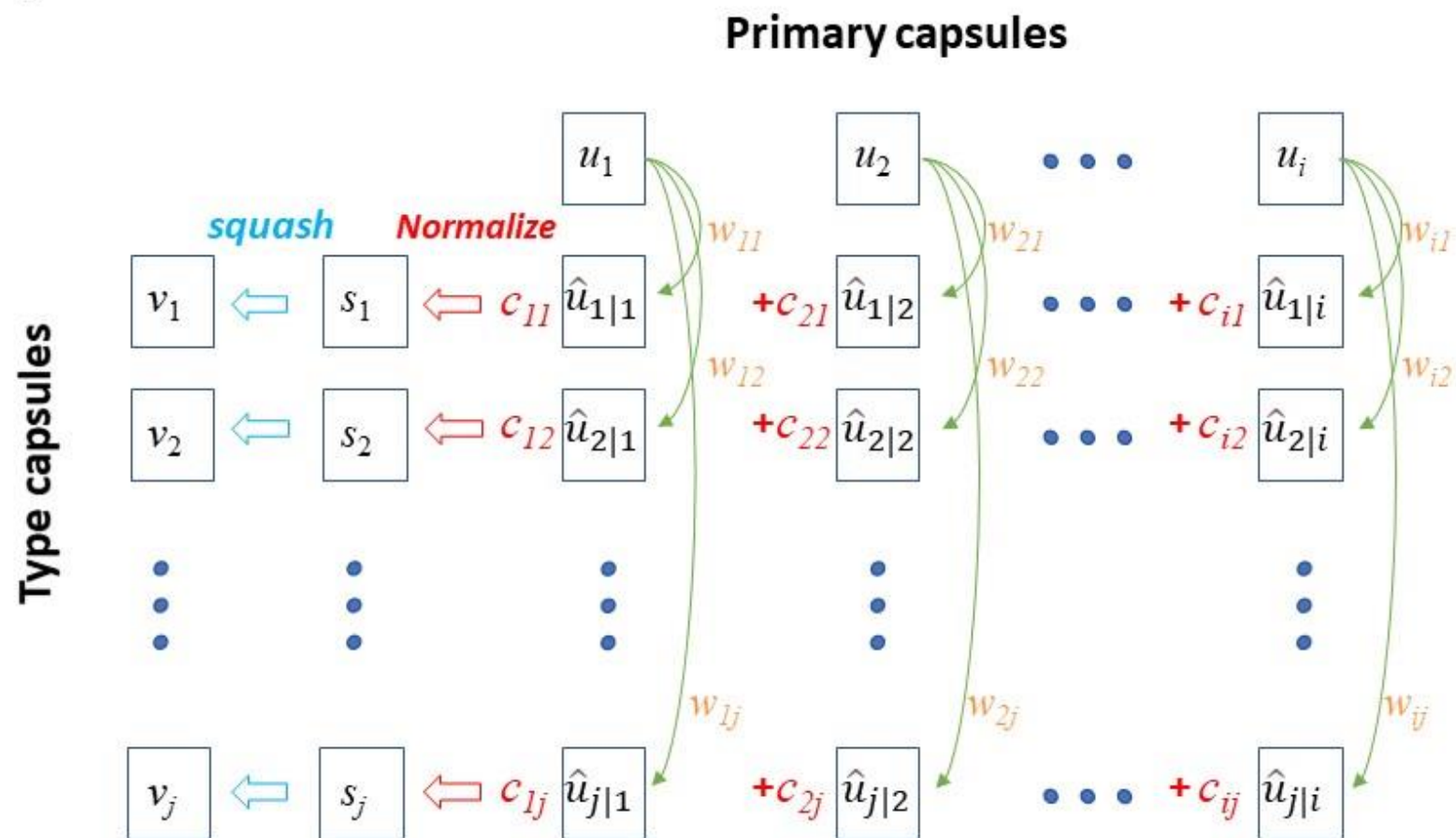

Figure S2

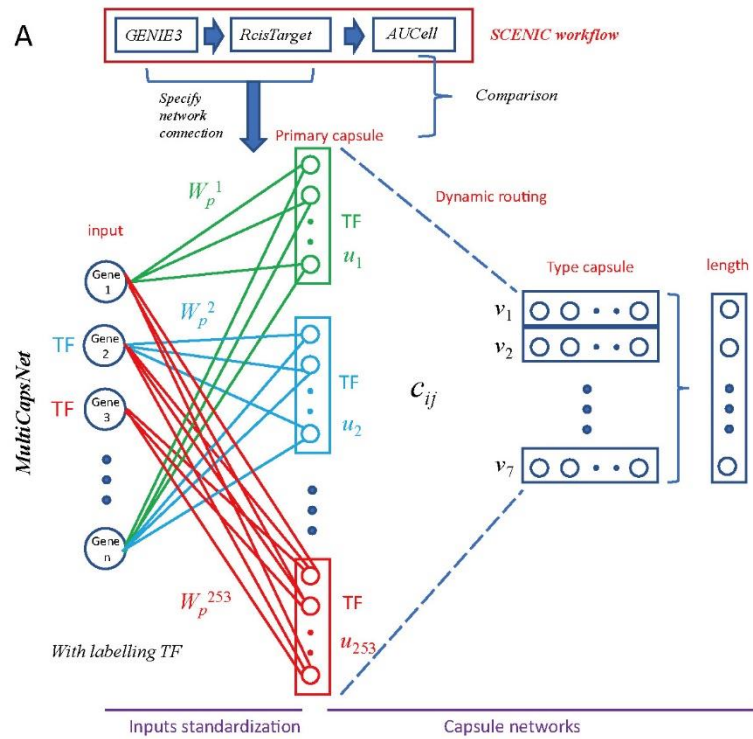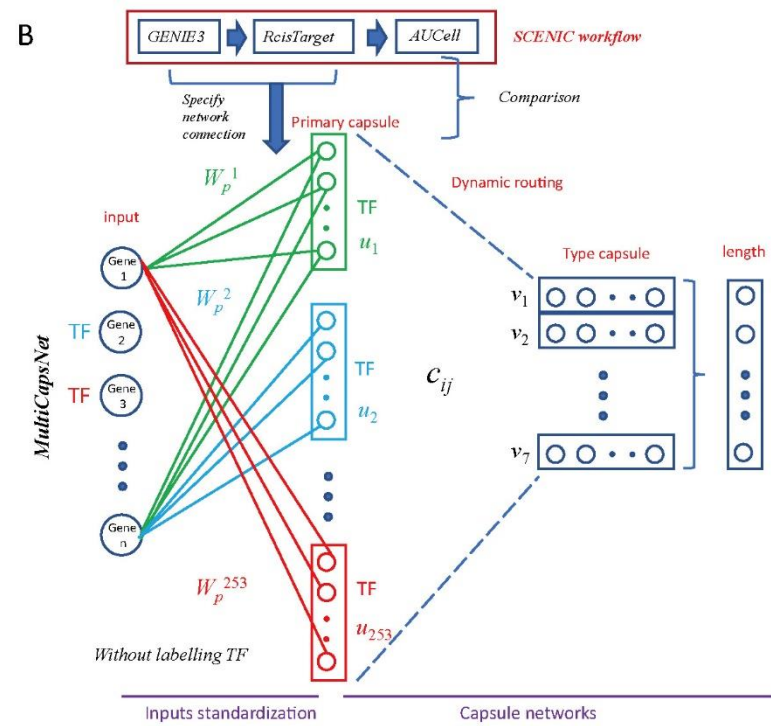

Figure S3

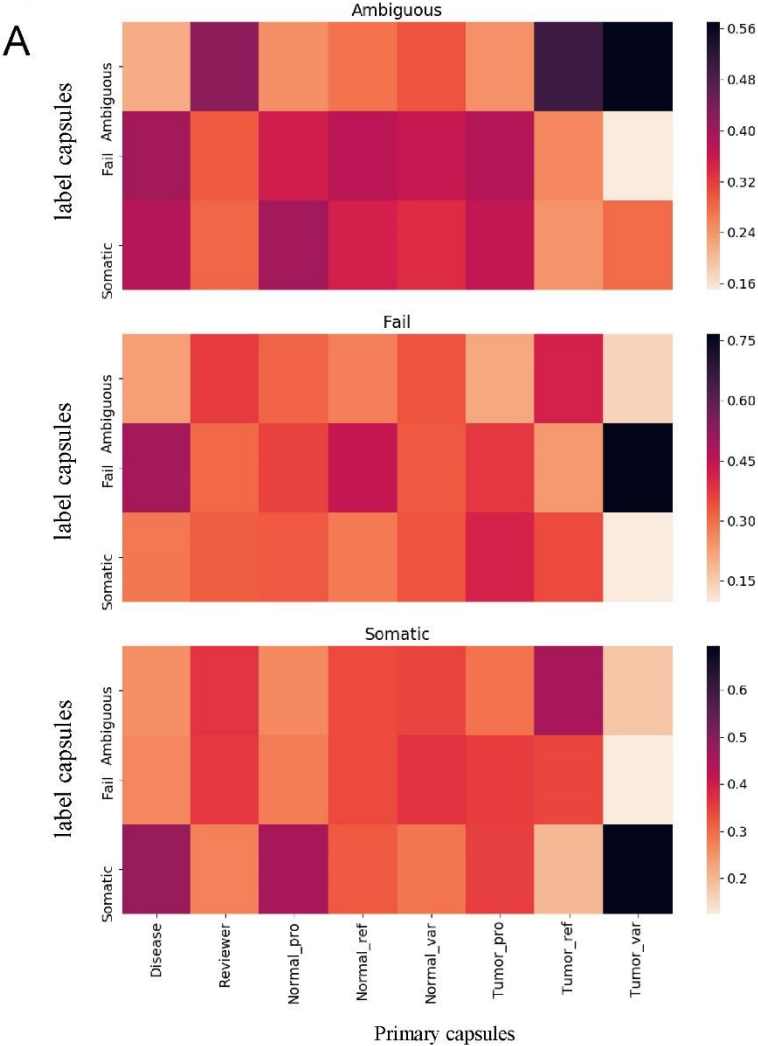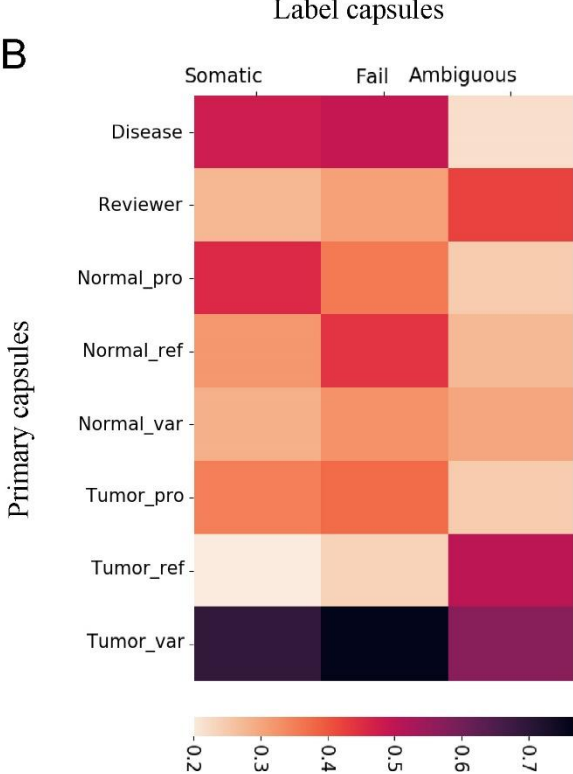

Figure S4

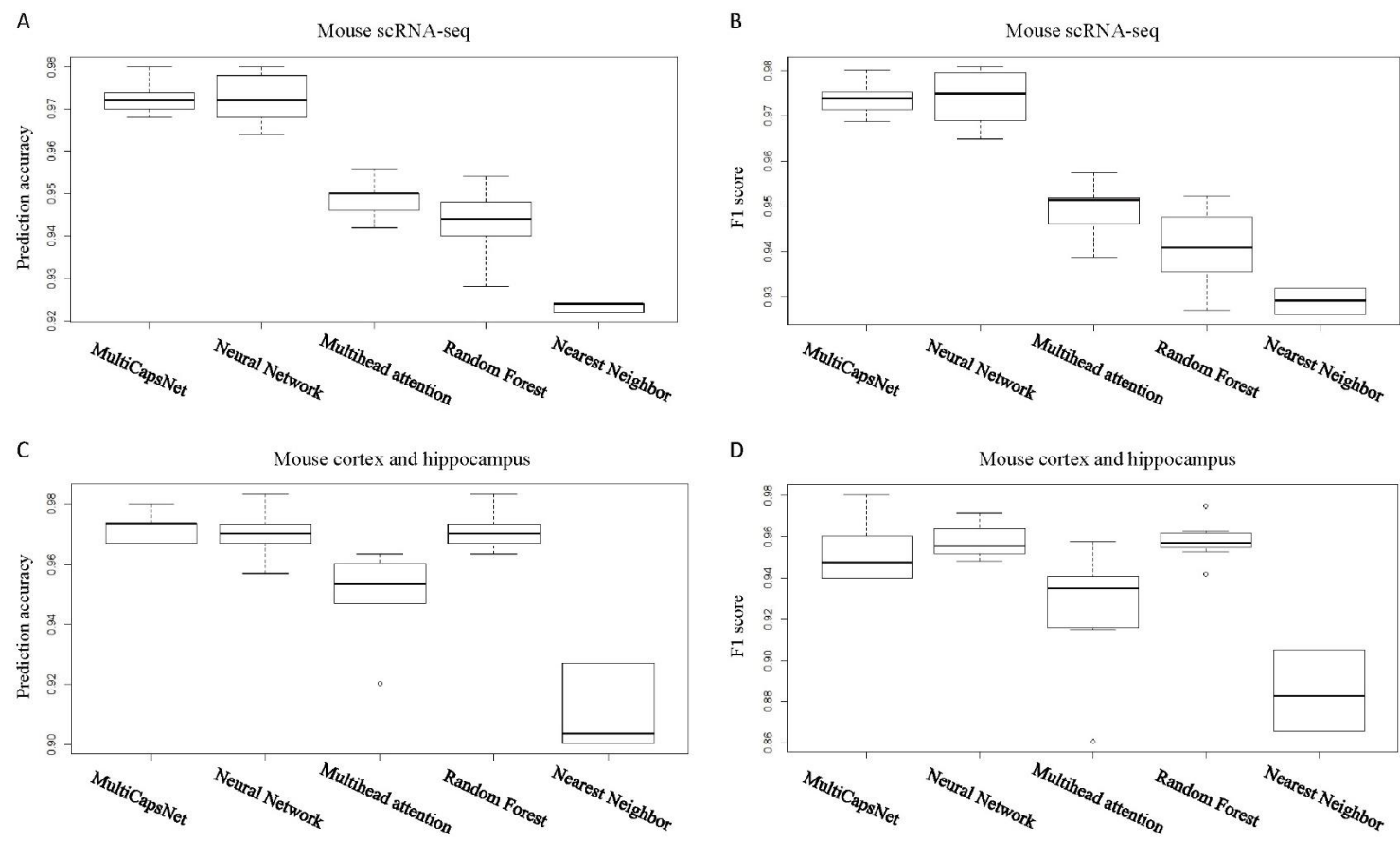

Figure S5

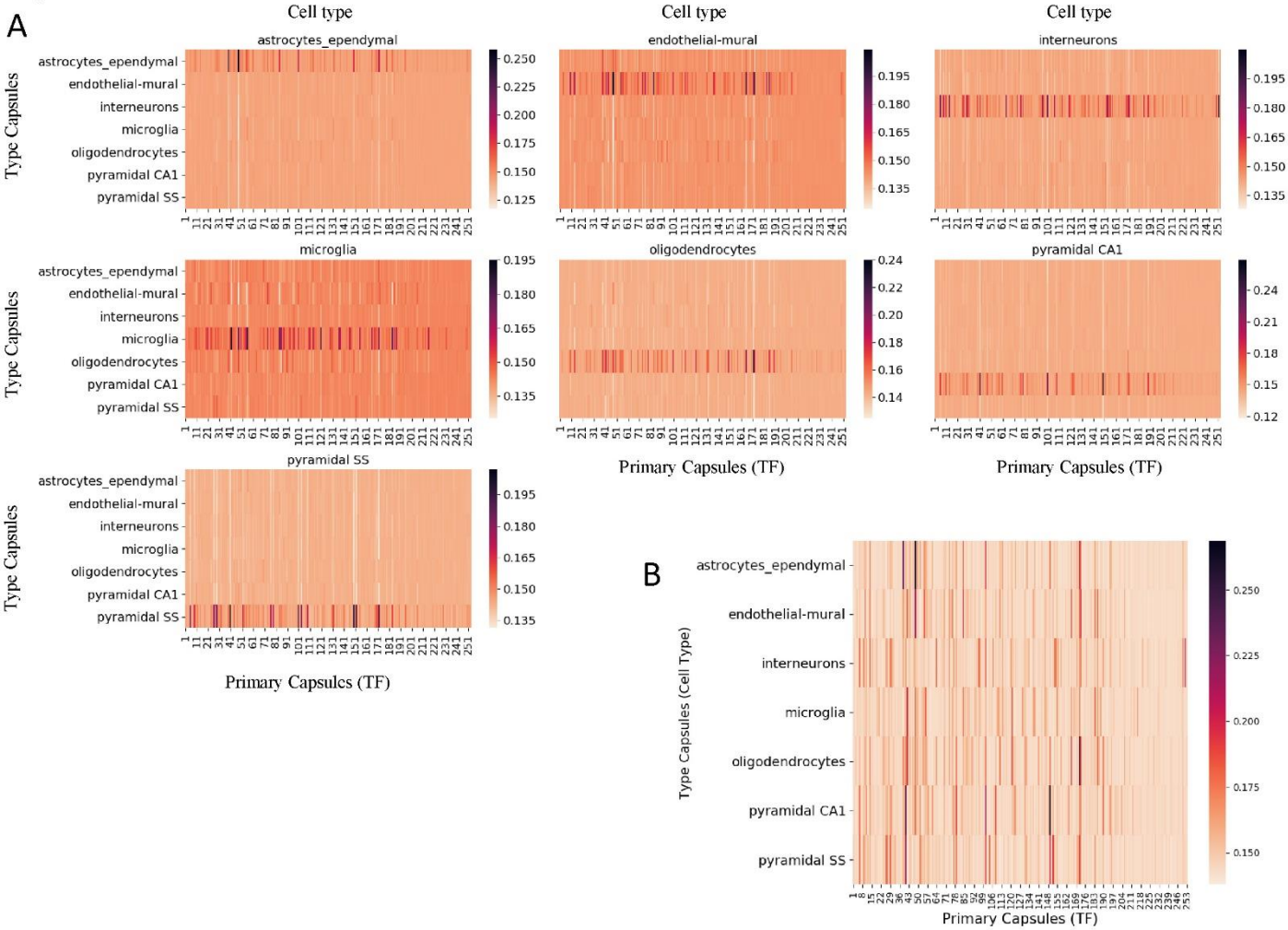

Supplement: Supplementary file 1 [file DataSheet2.PDF]
